# Augmenting pH Confers to *Citrus grandis* the Ability to Combat Oxidative Stress Triggered by Manganese Excess

**DOI:** 10.3390/plants15010172

**Published:** 2026-01-05

**Authors:** Rong-Yu Rao, Fei Lu, Bin-Bin Lan, Xian Zhu, Wei-Lin Huang, Xu-Feng Chen, Ning-Wei Lai, Lin-Tong Yang, Jiuxin Guo, Li-Song Chen

**Affiliations:** College of Resources and Environment, Fujian Agriculture and Forestry University, Fuzhou 350002, China; raorongyu00@163.com (R.-Y.R.); lzslf001@163.com (F.L.); lanbinbin2022@163.com (B.-B.L.); 15585832293@163.com (X.Z.); huangwl1821993@163.com (W.-L.H.); chengxufeng1996@163.com (X.-F.C.); lainingwei1109@fafu.edu.cn (N.-W.L.); talstoy@fafu.edu.cn (L.-T.Y.)

**Keywords:** ascorbate–glutathione cycle, glyoxalases, malondialdehyde, metallothioneins, methylglyoxal, phytochelatins, oxidative damage

## Abstract

Citrus trees are mainly cultivated in acidic soils. Excessive manganese (Mn) is the second most limiting factor for crop productivity in acidic soils after aluminum toxicity. The roles of reactive oxygen species (ROS) and methylglyoxal (MG) detoxification systems in augmented pH-mediated amelioration of excessive Mn are poorly understood. ‘Sour pummelo’ (*Citrus grandis* (L.) Osbeck) seedlings were exposed to nutrient solution at a Mn concentration of 500 (Mn500) or 2 (Mn2) μM and a pH of 3 (P3) or 5 (P5). The increase in pH attenuated Mn500-induced increases in ROS production and MG and malondialdehyde accumulation in roots and leaves. Additionally, the increase in pH enhanced the coordinated detoxification capability of both ROS and methylglyoxal scavenging systems in these tissues under Mn500. These findings corroborated the hypothesis that augmenting pH enhances the capability of these tissues to detoxify ROS and methylglyoxal under Mn excess. Therefore, this study provided new evidence on the roles of ROS and MG detoxification systems in the augmented pH-mediated amelioration of oxidative damage in ‘Sour pummelo’ leaves and roots caused by Mn excess, as well as a basis for correcting Mn toxicity by augmenting soil pH.

## 1. Introduction

Manganese (Mn) is both a micronutrient and a heavy metal (HM). When soil Mn^2+^ (phytoavailable form) is excessive, it is detrimental to crops [1,2,3]. Besides limiting crop productivity, excessive accrual of Mn^2+^ in soils may affect human health through the food chain [2]. Manganese excess typically occurs in crops grown in acidic soils with pH values below 5.0, as the concentration of Mn^2+^ in soil solution augments with reducing soil pH [1,4,5]. The soils in most citrus-producing areas are acidic due to soil acidification [5,6,7]. Manganese toxicity is the second most limiting factor in crop productivity on acidic soils after aluminum (Al) toxicity [8]. Wu et al. [6] reported that in 2374 soil samples and 2087 leaf samples from major citrus-growing regions in China, 49.1% of the soil samples had a pH value below 4.8 and 51.2% (47.5%) of the leaf (soil) samples had a Mn (available Mn) concentration above a sufficient range.

The resistance of plants to excessive Mn largely depends on soil acidity. Plants grown on acidic soils are often susceptible to Mn toxicity, while plants grown on alkaline soils are often prone to Mn deficiency [3]. Lime has traditionally been used to protect crops from the toxicity of Mn via augmenting soil pH [9,10]. A few studies have shown that augmenting pH can reduce Mn toxicity in plants. The reductions in white clover (*Trifolium repens* L.) and ryegrass (*Lolium perenne* L.) root and shoot dry weight (DW) caused by Mn toxicity was greater at pH 4.8 than at pH 6.0. There was no significant difference in the shoot Mn concentration at the two pH levels, whereas under Mn toxicity, the Mn concentration in ryegrass and white clover roots was lower and higher, respectively, at pH 4.8 than at pH 6.0 [11]. The amelioration of Mn toxicity in ‘Sour pummelo’ (*Citrus grandis* (L.) Osbeck) seedlings mediated by augmented pH was caused by a combination of factors, including (a) reduced Mn absorption; (b) less impairment to nutrient homeostasis; and (c) increased photosynthesis and growth [12]. Notably, most of these studies focused on the impacts of augmenting pH on plant growth, photosynthesis, and Mn and other nutrient homeostasis. However, the mechanism by which augmenting pH ameliorates Mn excess in plants is still unclear.

Excessive Mn can cause oxidative injury in plant cells via triggering the generation and over-accumulation of reactive oxygen species (ROS) and methylglyoxal (MG) [5,13,14,15]. The detoxification of ROS is carried out by the antioxidant defense system [15]. The system is composed of the ascorbate (ASC)–glutathione cycle, antioxidant enzymes, sulfur (S)-metabolizing enzymes, and S-containing compounds [13,16]. Activation of antioxidant enzymes and non-enzymatic antioxidants is considered to play a vital role in mitigating plant oxidative stress caused by excessive Mn [17,18]. Several antioxidant enzymes such as guaiacol peroxidase (EC 1.11.1.7, GuPX), superoxide dismutase (EC 1.15.1.1, SOD), and catalase (EC 1.11.1.6, CAT) and non-enzymatic antioxidants such as reduced glutathione (GSH), non-protein thiols (NP-SH), proline, and flavonoids were activated in ‘Fuji’ apple (*Malus domestica* Borkh.) seedlings by Mn excess to combat oxidative stress [2]. Excessive Mn increased the concentrations of non-enzymatic antioxidants and the activities of antioxidant enzymes in the Mn-tolerant stylo (*Stylosanthes guianensis* (Aubl.) Sw.) genotype but not in the Mn-sensitive genotype [4]. The tolerance of *M. hupehensis* (Pamp.) Rehd. to Mn excess conferred by H_2_S was associated with a lower Mn concentration in the stems, leaves, and roots, as well as lessened oxidative injury due to reduced accumulation of H_2_O_2_; augmented activities of monodehydroascorbate (MDHA) reductase (EC 1.6.5.4, MDHAR), CAT, GuPX, ASC peroxidase (EC 1.11.1.11, APX), SOD, and dehydroascorbate (DHA) reductase (EC 1.8.5.1, DHAR); and enhanced concentrations of GSH and ASC in leaves under Mn excess [14]. Phosphorus (P) supply decreased the concentrations of H_2_O_2_, superoxide anions, malondialdehyde (MDA), DHA, and oxidized glutathione (GSSG), while increasing the activities of GuPX, SOD, APX, CAT, MDHAR, and HHAR; concentrations of GSH and ASC; and ratios of GSG/GSSG and ASC/DHA in Mn-exposed peach (*Prunus persica* L.) leaves and roots [15]. A study from our laboratory showed that augmenting pH attenuated Mn excess-induced increases in Mn uptake and root and leaf Mn concentrations and decreases in S and P uptake and root and leaf S and P concentrations in ‘Sour pummelo’ seedlings [12]. This drives us to hypothesize that augmenting pH can enhance the plant’s ability to detoxify ROS under Mn excess by promoting P and S uptake and accumulation. The detoxification of MG is undertaken by glyoxalase I (EC 4.4.1.5, Gly I) and glyoxalase II (EC 3.1.2.6, Gly II) [19]. The coordinated effects of both ROS and MG detoxification systems play a role in the plant’s resilience to metals via combating oxidative stress brought about by metal toxicity [20]. Previous reports indicated that the mitigation of Al toxicity [16] and Cu excess [21] in ‘Xuegan’ (*Citrus sinensis* (L.) Osbeck) seedlings mediated by increased pH was associated with an augmented capability to detoxify ROS and MG in leaves and roots. This drives us to hypothesize that augmenting pH enhanced the capability of roots and leaves to detoxify ROS and MG under Mn excess. To our knowledge, very little is known about the impacts of augmenting pH on ROS and MG metabolisms in Mn-exposed plants. In a study, Rosas et al. [11] investigated the impacts of two pH levels (pH 4.8 and 6.0) and six Mn levels (0, 2.4 for ryegrass or 5.3 for white clover, 24, 59, 178, and 355 μM Mn) on the activities of GuPX in shoots. The activities of GuPX increased in response to high or low Mn concentrations at pH 4.8 and 6.0. The GuPX activity in ryegrass shoots was significantly higher at pH 4.8 than at pH 6.0 or similar between the two, but the reverse was the case in white clover shoots, with the exception that the GuPX activity at 5.3 μM Mn was significantly higher at pH 4.8 than at pH 6.0. Unfortunately, the study did not assay the activities of other enzymes associated with ROS and MG detoxification and the concentrations of non-enzymatic antioxidants. Collectively, the roles of ROS and MG detoxification systems in augmented pH-mediated amelioration of excessive Mn are poorly understood.

To date, detailed studies have been conducted on the impacts of low pH or Mn excess on citrus at physiological and molecular scales [5,21,22,23,24,25,26,27]. There is currently no research on the impacts of Mn–pH treatments on ROS and MG metabolisms in citrus. In this study, we investigated the influences of Mn–pH treatments on ROS and MG detoxification and their generation in roots and leaves of ‘Sour pummelo’ seedlings. The aim was to corroborate the hypothesis that augmenting pH enhanced the capability of roots and leaves to detoxify ROS and MG under Mn excess.

## 2. Results

### 2.1. Augmenting pH Attenuated Excessive Mn-Induced Changes in ROS Production, MG, MDA, S-Containing Compounds, and Antioxidant Concentrations in Roots and Leaves

**Figure 1:**
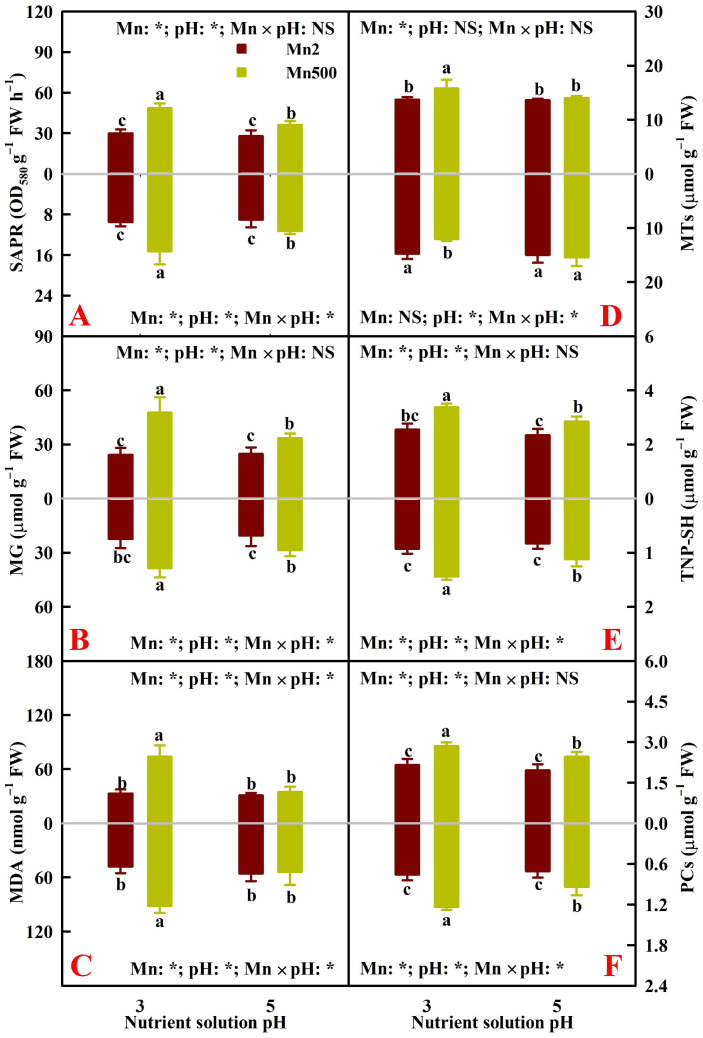
Effects of Mn–pH treatments on the mean (±SD, *n* = 4) SAPR (**A**) and concentrations of MDA (**B**), MG (**C**), MTs (**D**), TNP-SH (**E**), and PCs (**F**) in roots (below column) and leaves (above column). The bars with different letters indicate significant differences at *p* ≤ 0.05. pH: NS and Mn × pH: NS indicate that the *F* values for pH and Mn × pH are not significant (*p* > 0.05). Mn: *, pH: *, and Mn × pH: * indicate that the *F* values for Mn, pH, and Mn × pH are significant at *p* ≤ 0.05.

The research showed that 500 μM Mn (Mn500 or excessive Mn) significantly increased root and leaf MG concentrations by 71.3% and 96.9%, MDA concentrations by 91.6% and 124.4%, and superoxide anion production rate (SAPR) by 60.8% and 62.1%, respectively, under pH 3 (P3) at *p* ≤ 0.05, as well as root and leaf MG concentrations by 39.5% and 35.3% and SAPR by 24.2% and 29.8%, respectively, under pH 5 (P5) at *p* ≤ 0.05; however, Mn500 did not significantly alter root and leaf MDA concentrations under P5 (*p* > 0.05; Figure 1A–C).

The study showed that Mn500 significantly increased root and leaf phytochelatin (PC) concentrations by 62.9% and 32.8% and total non-protein thiol (TNP-SH) concentrations by 55.4% and 32.2%, respectively, under P3, as well as root and leaf PC concentrations by 32.6% and 25.3% and TNP-SH concentrations by 35.3% and 22.2%, respectively, under P5; and that Mn500 significantly decreased and increased metallothionein (MT) concentrations in roots and leaves, respectively, under P3 but not under P5 (Figure 1D–F). The different responses of root and leaf MTs to Mn500 agreed with the reports that lead (Pb) toxicity downregulated and upregulated *MT3* expression in tomato (*Solanum lycopersicum* L.) roots and leaves, respectively [28], and that after exposure to 0.5 and 1 mM zinc (Zn), the expression level of *MT2* decreased in white poplar (*Populus alba* L.) roots but increased in its leaves [29]. These results suggested that the levels of MTs might be differentially altered by Mn500 in an organ-specific manner.

**Figure 2:**
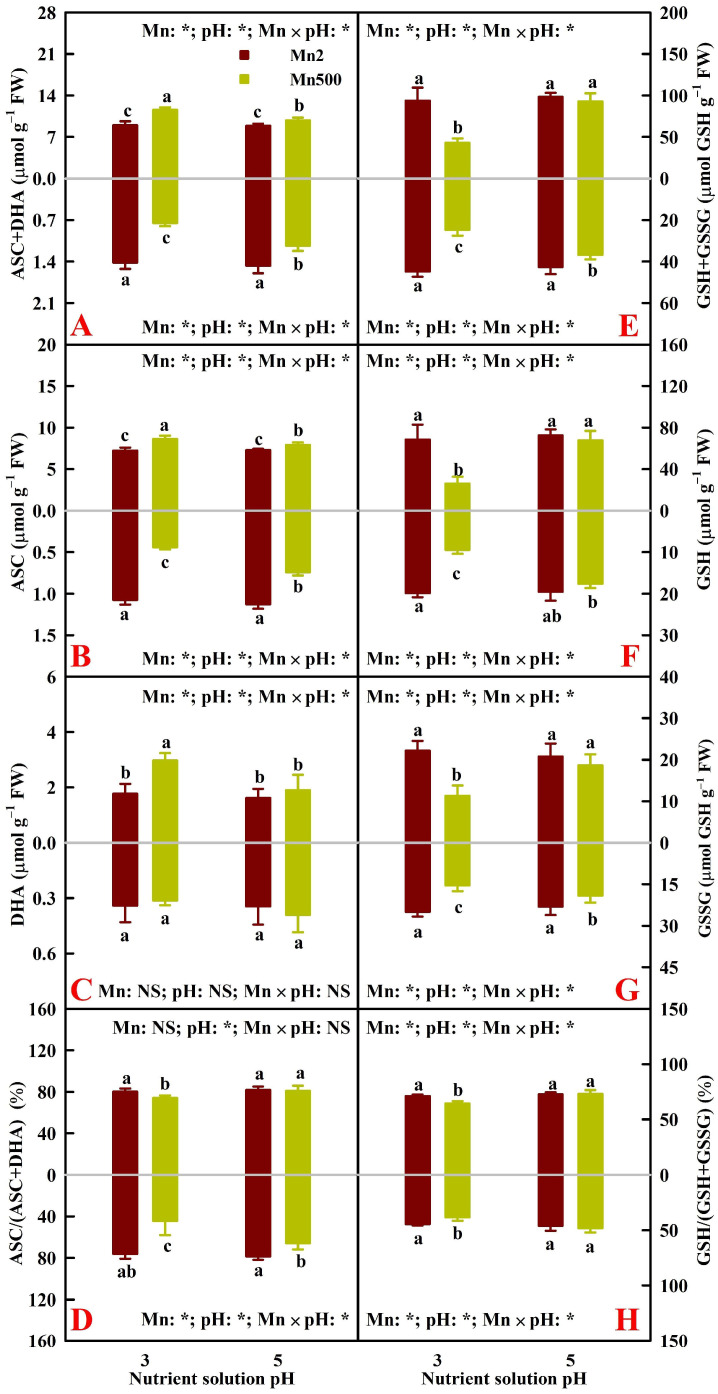
Effects of Mn–pH treatments on the mean (±SD, *n* = 4) ASC+DHA (TA; (**A**)), ASC (**B**), and DHA (**C**) concentrations; ASC/TA ratios (**D**); GSH+GSSG (TG; (**E**)), GSH (**F**), and GSSG (**G**) concentrations; and GSH/TG ratios (**H**) in roots (below column) and leaves (above column). The bars with different letters indicate significant differences at *p* ≤ 0.05. Mn: NS, pH: NS, and Mn × pH: NS indicate that the *F* values for Mn, pH, and Mn × pH are not significant (*p* > 0.05). Mn: *, pH: *, and Mn × pH: * indicate that the *F* values for Mn, pH, and Mn × pH are significant at *p* ≤ 0.05.

The current research showed that Mn500 significantly increased leaf ASC, DHA, and total ascorbate (TA, DHA+ASC) concentrations by 19.6% (8.7%; values in parentheses correspond to P5), 67.8% (17.3%), and 29.1% (10.3%) under P3 (P5), respectively; decreased root ASC and TA concentrations by 59.0% (34.0%) and 46.8% (22.9%) under P3 (P5), respectively; and did not significantly alter root DHA concentrations under P3 and P5. A greater increase in the TA level than in the ASC level in P3-exposed leaves in response to Mn500 led to a significant decrease in ASC/TA, whereas a greater decrease in the ASC level than in the TA level in roots in response to Mn500 caused a significant decrease in ASC/TA, especially under P3 (Figure 2A–D). This agreed with the report that TiO_2_ nanoparticle toxicity increased leaf TA concentration and did not alter root TA concentration in wheat (*Triticum aestivum* L.) seedlings [30].

The research showed that Mn500 significantly decreased leaf (root) GSH, oxidized glutathione (GSSG), and total glutathione (TG, GSH+GSSG) concentrations by 62.1% (52.3%; values in parentheses correspond to roots), 49.2% (38.6%), and 54.3% (44.7%) under P3, respectively, as well as root GSSG and TG by 17.1% and 13.8% under P5, respectively; however, Mn500 did not significantly alter leaf GSH, GSSG, and TG concentrations and root GSH concentrations. A greater decrease in the GSH level than in the TG level in P3-exposed leaves and roots in response to Mn500 led to a significant decrease in GSH/TG (Figure 2E–H). This agreed with the report that H_2_S-mediated mitigation of oxidative stress caused by Cr(VI) in black bean (*Vigna mungo* L.) and mung bean (*Vigna radiata* L.) leaves and roots involved an increase in GSH/GSSG caused by a greater increase in GSH concentration than in GSSG concentration [31].

Notably, the values for the leaf and root ASC, DHA, TA, GSH, GSSG, and TG concentrations and ASC/TA and GSH/TG ratios obtained here were comparable to the values for these parameters reported previously on cucumber (*Cucumis sativus* L.) [32] and *Nitraria tangutorum* Bobr. [33].

### 2.2. Impacts of Mn–pH Treatments on the Activities of Enzymes for the Detoxification of ROS and MG in Roots and Leaves

**Figure 3:**
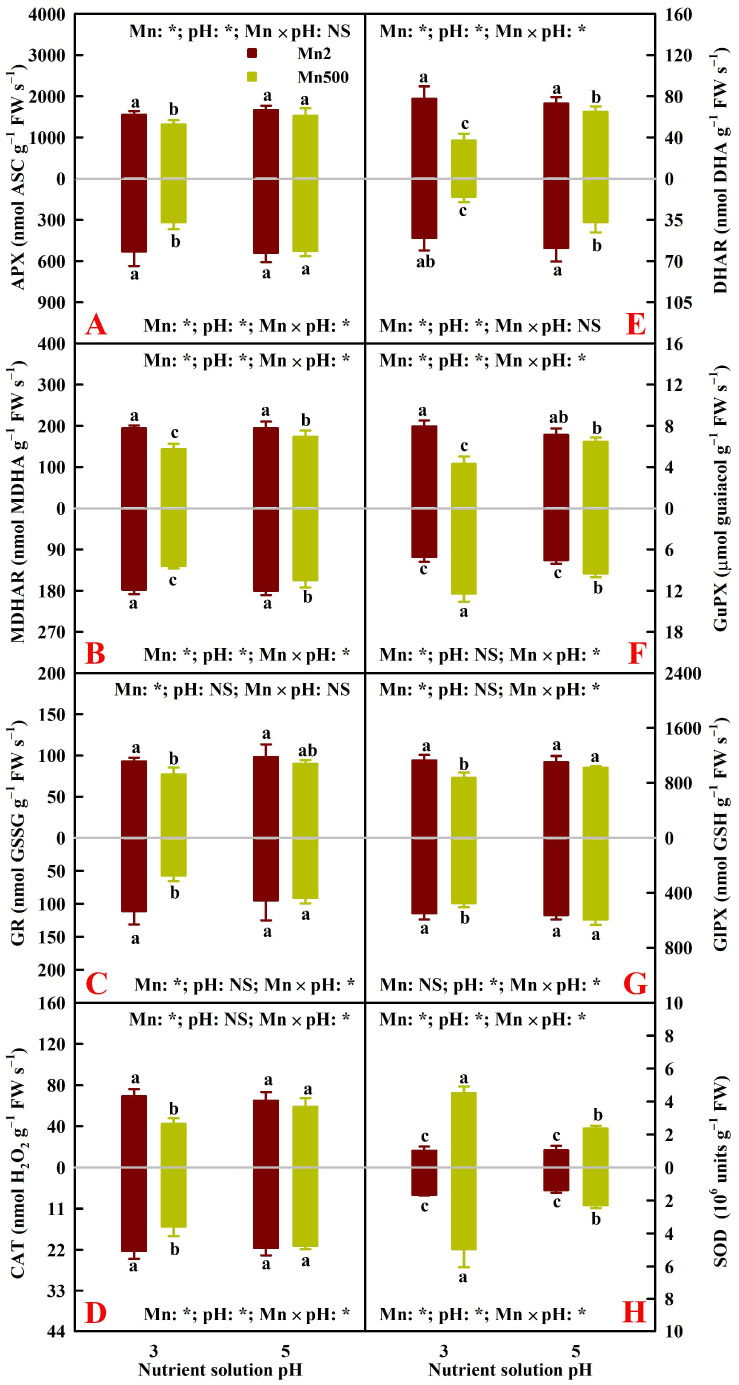
Effects of Mn–pH treatments on the mean (±SD, *n* = 4) activities of APX (**A**), MDHAR (**B**), GR (**C**), CAT (**D**), DHAR (**E**), GuPX (**F**), GlPX (**G**), and SOD (**H**) in roots (below column) and leaves (above column). The bars with different letters indicate significant differences at *p* ≤ 0.05. Mn: NS, pH: NS, and Mn × pH: NS indicate that the *F* values for Mn, pH, and Mn × pH are not significant (*p* > 0.05). Mn: *, pH: *, and Mn × pH: * indicate that the *F* values for Mn, pH, and Mn × pH are significant at *p* ≤ 0.05.

Figure 3 reveals the impacts of Mn–pH treatments on the activities of ASC–glutathione cycle enzymes (APX, MDHAR, HDAR, and glutathione reductase (EC 1.6.4.2, GR)) and other antioxidant enzymes (SOD, CAT, glutathione peroxidase (EC 1.11.1.9, GlPX), and GuPX) in leaves and roots. The increase in pH attenuated Mn500-induced decreases in the activities of root and leaf ASC–glutathione cycle enzymes, CAT, and GlPX, as well as leaf GuPX, but it attenuated Mn500-triggered increases in the activities of root and leaf SOD and root GuPX. This agreed with the previous reports that Mn excess decreased the activities of four ASC–glutathione cycle enzymes and CAT but increased the activities of SOD in peach roots and leaves [15] and that Zn excess increased and decreased GuPX activities in wheat leaves and roots, respectively [34].

**Figure 4:**
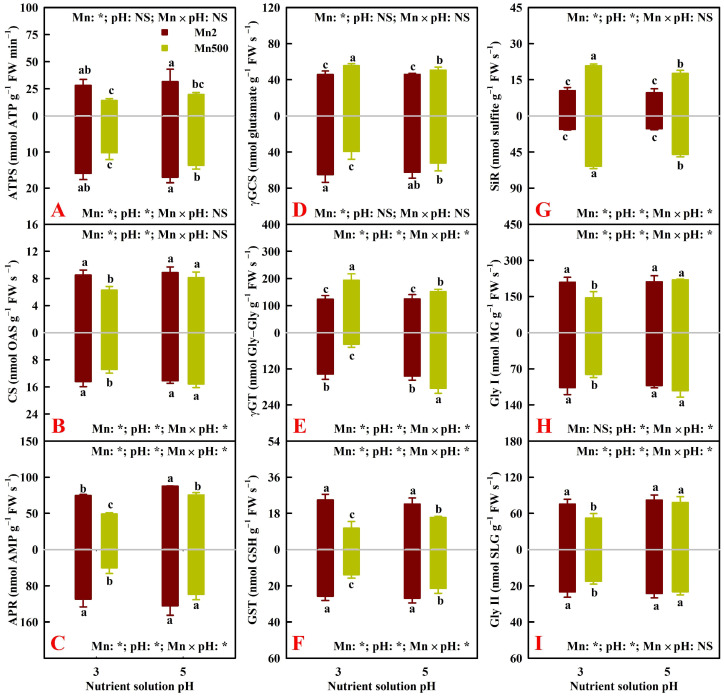
Impacts of Mn–pH treatments on the mean (±SD, *n* = 4) activities of ATPS (**A**), CS (**B**), APR (**C**), γGCS (**D**), γGT (**E**), GST (**F**), SiR (**G**), Gly I (**H**), and Gly II (**I**) in roots (below column) and leaves (above column). The bars with different letters indicate significant differences at *p* ≤ 0.05. Mn: NS, pH: NS, and Mn × pH: NS indicate that the *F* values for Mn, pH, and Mn × pH are not significant (*p* > 0.05). Mn: *, pH: *, and Mn × pH: * indicate that the *F* values for Mn, pH, and Mn × pH are significant at *p* ≤ 0.05. OAS, O-acetylserine.

Figure 4A–G exhibit the impacts of Mn–pH treatments on seven S-metabolizing enzyme activities. The increase in pH mitigated Mn500-triggered decreases in root and leaf ATP sulfurylase (EC 2.7.7.4, ATPS), cysteine synthase (EC 2.5.1.47, CS), adenosine 5′-phosphosulphate (APS) reductase (EC 1.8.99.2, APR), and glutathione S-transferase (EC 2.5.1.18, GST) activities and root γ-glutamylcysteine synthetase (EC 6.3.2.2, γGCS) and γ-glutamyltransferase (EC 2.3.2.2, γGT) activities, as well as increases in root and leaf sulfite reductase (EC 1.8.1.2, SiR) activities and leaf γGCS and γGT activities. Ahmad et al. [35] observed that drought stress in maize (*Zea mays* L.) led to differential acclimation responses of S metabolism in roots and leaves. Cadmium (Cd) downregulated the expression of the cysteine synthase 26 gene in sorghum (*Sorghum bicolor* (L.) Moench) leaves but not in roots and upregulated the expression of *ATPS1* in roots but not in leaves [36]. NaCl stress upregulated and downregulated *ATPS1* expression in sorghum leaves and roots, respectively [37]. These results implied that the activities of γGCS and γGT might be differentially affected by Mn500 in an organ-specific manner.

The current research showed that Mn500 lowered Gly I and Gly II activities in roots and leaves at P3 but not at P5 (Figure 4H,I).

To conclude, the increase in pH lessened Mn500-triggered alterations in the activities of root and leaf antioxidant enzymes, S-metabolizing enzymes, and glyoxalases.

### 2.3. Principal Coordinate Analysis (PCoA), Regression Analysis, and Different Responses of Root and Leaf ROS and MG Formation and Detoxification to Mn–pH Treatments

**Figure 5:**
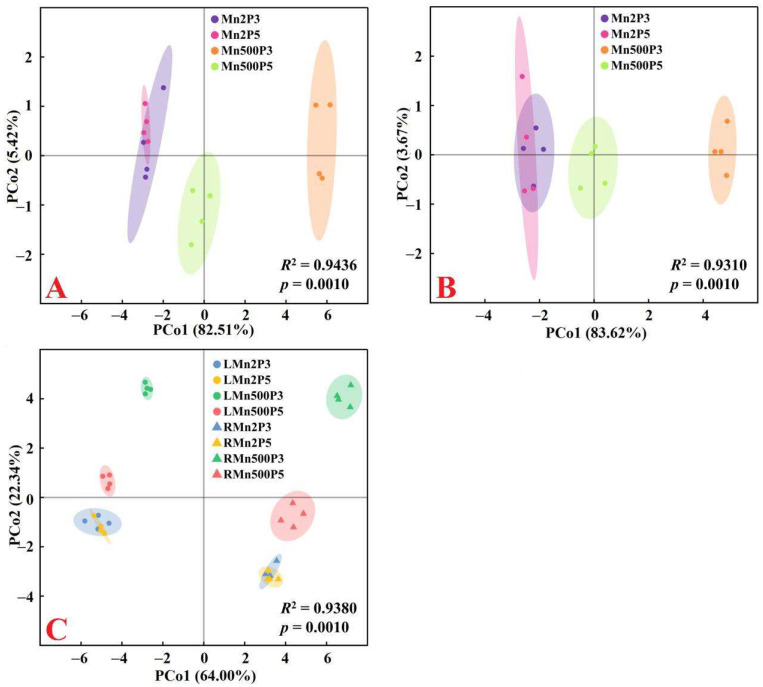
Principal coordinate analysis plots of 31 parameters for roots (**A**), leaves (**B**), and both roots and leaves (**C**) from ‘Sour pummelo’ seedlings exposed to 2 (Mn2) or 500 (Mn500) μM Mn and a pH of 3 (P3) or 5 (P5). Data from Figure 1, Figure 2, Figure 3 and Figure 4. RMn2P5, roots of Mn2 + P5-treated seedlings; RMn2P3, roots of Mn2 + P3-treated seedlings; RMn500P5, roots of Mn500 + P5-treated seedlings; RMn500P3, roots of Mn500 + P3-treated seedlings; LMn2P5, leaves of Mn2 + P5-treated seedlings; LMn2P3, leaves of Mn2 + P3-treated seedlings; LMn500P5, leaves of Mn500 + P5-treated seedlings; LMn500P3, leaves of Mn500 + P3-treated seedlings. Clustering of samples with PCoA was performed based on the Canberra distance matrix. PERMANOVA was applied to test the significance of separation. The ellipse represents the 95% confidence interval around the centroid of each group.

A PCoA was made using the 31 indexes, including 17 enzymes, 8 antioxidants and ratios, SAPR, MG, MDA, MTs, PCs, and TNP-SH in roots (Figure 5A), leaves (Figure 5B), and both roots and leaves (Figure 5C). It was shown that the first two components contributed to 87.93% (82.51% for PCo1 and 5.42% for PCo2), 87.29% (83.62% for PCo1 and 3.67% for PCo2), and 86.34% (64.00% for PCo1 and 22.34% for PCo2) of the total variation in roots, leaves, and both roots and leaves, respectively, and that the differences between groups were significant in roots, leaves, and both roots and leaves (Figure 5). For roots or leaves, PCo1 indicated clear Mn-toxic impacts and low pH effects at Mn500 (Figure 5A,B). The shorter distance between Mn2+P5 (Mn2P5) and Mn500+P5 (Mn500P5) than between Mn2+P3 (Mn2P3) and Mn500+P3 (Mn500P3) (Figure 5A,B) suggested that the increase in pH reduced the changes in these 31 root and leaf parameters caused by Mn500. This agreed with the data that Mn500 did not significantly change eleven (two indexes from Figure 1, three indexes from Figure 2, four indexes from Figure 3, and two indexes from Figure 4) out of the thirty-one root indexes at P5 but only one (one index from Figure 2) out of thirty-one root indexes at P3, as well as sixteen (two indexes from Figure 1, six indexes from Figure 2, five indexes from Figure 3, and three indexes from Figure 4) out of thirty-one leaf indexes at P5 and zero out of thirty-one leaf indexes at P3.

**Figure 6:**
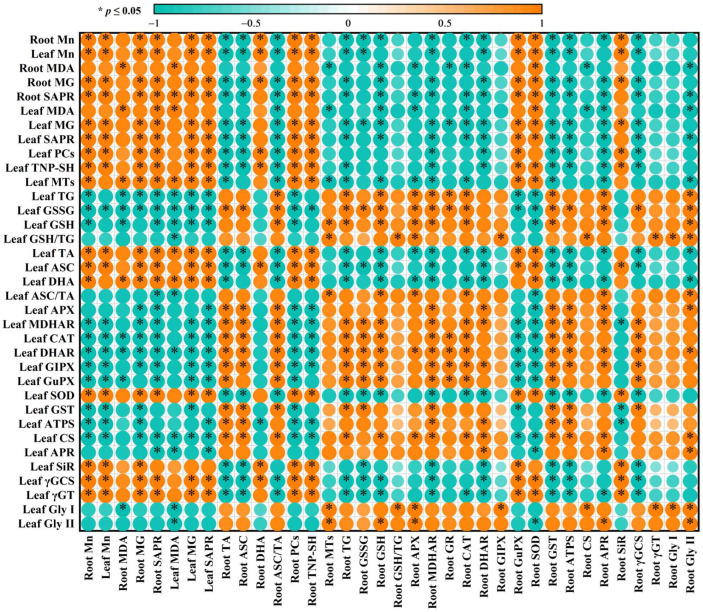
A Pearson’s correlation coefficient matrix for the mean valves of some indexes (*n* = 4) in roots and leaves. Manganese concentrations for roots and leaves from [12]; other data from Figure 1, Figure 2, Figure 3 and Figure 4. *, significant at *p* ≤ 0.05.

**Figure 7:**
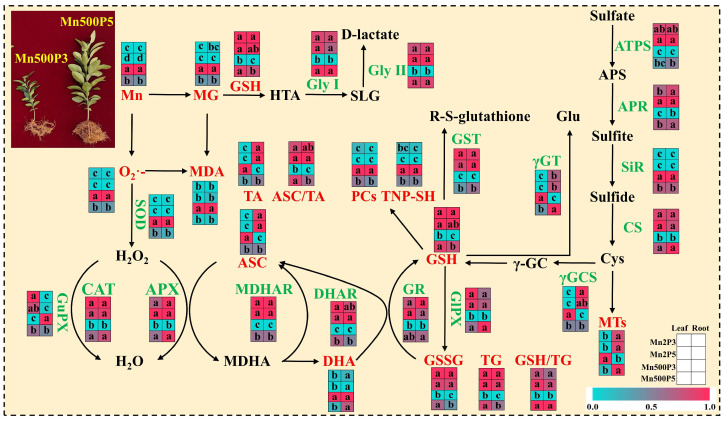
A diagram displaying the raised pH-mediated alleviation of oxidative stress in ‘Sour pummelo’ roots and leaves. Manganese concentrations for roots and leaves from [12]; other data from Figure 1, Figure 2, Figure 3 and Figure 4. For roots or leaves, different letters indicate significant differences at *p* ≤ 0.05.

For both roots and leaves, PCo1 clearly separated the roots (clustered on the right side) from the leaves (clustered on the left side) (Figure 5C). This suggested that the responses of the 31 parameters to Mn–pH treatments differed between roots and leaves, which was supported by our data that five leaf indexes (GuPX, γGCS, MTs, TA, and ASC) displayed a significant negative correlation with the corresponding root indexes (Figure 6 and Table S1); that Mn500 increased and did not alter DHA concentrations at P3 in leaves and roots, respectively (Figure 2C); and that Mn500 increased and decreased γGT activities at P3 in leaves and roots, respectively (Figure 4E). Additionally, 19 leaf indexes (MDA, MG, SAPR, PCs, TNP-SH, MTs, TG, GSSG, GSH, GSH/TG, MDHAR, CAT, SOD, GST, ATPS, APR, SiR, Gly I, and Gly II) displayed a significant positive correlation with the corresponding root indexes (Figure 6 and Table S1) and 6 indexes (ASC/TA, APX, GR, DHAR, GlPX, and CS) displayed similar responses to Mn–pH treatments in leaves and roots (Figure 2, Figure 3 and Figure 4). To conclude, the responses of indexes associated with ROS and MG formation and removal to Mn–pH treatments differed between roots and leaves (Figure 7).

## 3. Discussion

The research indicated that in roots and leaves, MG concentration and SAPR were significantly positively related with the Mn level, and the MDA level displayed an augmented trend with increasing MG level or SAPR (Figure 6), implying that augmenting pH mitigated Mn500-triggered generation and accumulation of ROS and MG due to lessened Mn concentration in roots and leaves, thereby contributing to less MDA accumulation under Mn500 (Figure 1).

The ascorbate–glutathione cycle (mainly composed of ASC, GSH, APX, GR, MDHAR, and DHAR) and other antioxidant enzymes (CAT, SOD, GlPX, and GuPX) function in the removal of ROS [13,16]. The study showed that the increase in pH reduced Mn500-triggered alterations in the activities of eight antioxidant enzymes (Figure 3); the concentrations of GSH, GSSG, TG, ASC, DHA, and TA; and the ratios of ASC/TA and GSH/TG in roots and leaves, except for root DHA concentration (Figure 2). The thiol-based antioxidant system also plays a key role in the scavenging of ROS [16]. The research revealed that increasing pH attenuated Mn500-triggered changes in the activities of seven S-metabolizing enzymes (Figure 2) and the concentrations of MTs, PCs, and TNP-SH in roots and leaves (Figure 1). In leaves, SAPR declined significantly with increasing APX, GR, MDHAR, DHAR, CAT, GlPX, GuPX, ATPS, CS, or APR activity and TG, GSH, or GSSG concentration and showed a decreased trend with increasing GST activity and GSH/TG or ASC/TA. However, SAPR increased significantly with increasing SOD, γGCS, or γGT activity and PC, TNP-SH, MT, TA, ASC, or DHA concentration and showed an increased trend with increasing SiR activity (Figure 6 and Table S1). In roots, SAPR declined significantly with increasing APX, MDHAR, DHAR, CAT, GST, APR, ATPS, or γGCS activity; TA, ASC, TG, or GSH concentration; and ASC/TA and showed a reduced trend with increasing GR, GlPX, CS, or γGT activity; MT or GSSG concentration; and GSH/TG. However, SAPR increased significantly with increasing GuPX or SOD activity and PC or TNP-SH concentration and showed an increased trend with increasing SiR activity and DHA concentration (Figure 6 and Table S1). One of the adaptive alterations to alleviate Mn excess in plants involves activating the antioxidant system through the antioxidant enzymes and antioxidants (GSH and ASC) [38]. Manganese excess activated the antioxidant scavenging system in the Mn-tolerant stylo genotype but not in the Mn-sensitive one [4]. Ascorbate, GSH, PCs, and MTs may chelate metal ions, thus detoxifying metal ions and reducing their catalytic activity in forming ROS [39,40]. In addition to acting as a chelating agent to detoxify HMs, GSH can also activate GST activity to protect the membrane from harmful effects of substances produced by lipid and protein oxidation [41]. Cellular redox status (ASC/TA and GSH/TG) may play a more important role in the defense response against ROS detoxification than ASC and/or GSH levels [42]. Ascorbate addition (≤50 mg L^−1^) ameliorated Mn excess-induced oxidative stress in *Vallisneria natans* (Lour.) Hara through lowering Mn excess-induced accumulation of H_2_O_2_; counteracting Mn excess-induced decrements of CAT, APX, and SOD activities; and increasing GuPX activity [43]. Augmented pH-mediated mitigation of Cu excess and Al toxicity in ‘Xuegan’ roots and leaves involved a decrease in the formation and accumulation of ROS, an upregulation in the ROS detoxification system, and an increase in the GSH/TG and ASC/TA [16,21]. These results suggested that in addition to lowering the Mn500-stimulated formation of ROS, the increase in pH mitigated the damage of Mn500 to the ROS detoxification system (ASC–glutathione cycle, other antioxidant enzymes, and thiol-based antioxidant system), thereby lowering root and leaf accumulation of ROS and protecting them from oxidative injury. Our findings that Mn500 significantly increased root and leaf PC and TNP-SH concentrations and SOD and SiR activities, leaf MT and ASC concentrations, leaf γGCS and γGT activities, and root GuPX activity under P3, but to a lower extent or not at all under P5 (Figure 7), agreed with the more increased demand for ROS detoxification (Figure 1A). However, these adaptive responses did not protect P3-exposed roots and leaves from oxidative damage caused by Mn500.

Noor et al. [15] reported that P application could effectively reduce excessive Mn-induced oxidative injury in peach roots and leaves by lowering H_2_O_2_ accumulation, elevating the activities of ASC–glutathione cycle enzymes, CAT, and SOD and maintaining the cellular redox status (ASC/TA and GSH/TG) due to decreased DHA and GSSG concentrations and increased ASC and GSH concentrations. Liang et al. [44] showed that S supply promoted S assimilation and glutathione metabolism in Cd-stressed pakchoi (*Brassica chinensis* L.) roots and leaves, thereby attenuating Cd toxicity-triggered oxidative damage in roots and leaves. Melatonin-mediated mitigation of oxidative stress in tomato leaves and roots involved enhanced S uptake (concentration) and assimilation as well as antioxidant enzyme activities and antioxidant (GSH) concentration [45]. Rao et al. [12] indicated that augmenting pH alleviated Mn500-triggered reductions in leaf and root P and S concentrations and P and S uptake in ‘Sour pummelo’ seedlings. These results suggested that the increase in P and S uptake caused by augmented pH played a role in protecting roots and leaves from oxidative stress under Mn500.

The research indicated that the increase in pH attenuated Mn500-induced decreases in root and leaf GSH and GSSG concentrations, especially the GSH concentrations, thereby enhancing the root and leaf GSH and GSSG concentrations and GSH/TG under Mn500 (Figure 2). This was supported by the results that H_2_S attenuated Cr(VI)-induced oxidative damage in black bean and mung bean leaves and roots but increased their GSH and GSSG concentrations, especially the GSH concentration, thereby enhancing GSH/GSSG in roots and leaves exposed to Cr(VI) [31]. Ostaszewska-Bugajska et al. [46] reported that S deficiency reduced root and leaf GSH and GSSG concentrations except for an unaltered root GSSG concentration in *Arabidopsis thaliana* (L.) Heynh. seedlings. The increases in S uptake and root and leaf S concentrations caused by increased pH might contribute to the higher leaf and root GSH and GSSG concentration at P5 than at P3 under Mn500 (Figure 2). These results suggested that the increase in pH might enhance the ability to maintain the glutathione redox state and glutathione in leaves and roots, thereby protecting them from oxidative damage under Mn500. The higher GSSG level in Mn-exposed leaves and roots under P5 than under P3 did not necessarily imply that Mn500-triggered oxidative damage in leaves and roots was more severe under P5 than under P3, because relatively less GSH was oxidized to GSSG, as evidenced by the higher GSH/TG (Figure 2H).

In plant cells, GSH concentration depends on its biosynthesis, catabolism (degradation), utilization, and regeneration. Adenosine 5′-phosphosulphate is reduced to sulfite by APR. Sulfite is further reduced by SiR to sulfide, which is then incorporated into O-acetylserine (OAS) by CS to yield cysteine. Cysteine can serve as a precursor for the synthesis of PCs and GSH [47]. Enhanced utilization of GSH for PC biosynthesis at HM exposure can transiently lower the GSH level [48]. γ-Glutamylcysteine synthetase catalyzes the de novo biosynthesis of GSH [49]. The yielded GSH can be used for various redox reactions to prevent plant cells from oxidative injury, while GSH is oxidized to GSSG, which is then regenerated by GR to GSH [42]. Ding et al. [50] found that under normal conditions, the ratios of ASC/DHA and GSH/GSSG and the levels of DHA, ASC, GSH, and GSSG were similar between wild type (WT) and transgenic tobacco leaves with 30–70% WT leaf GR activity. Under oxidative stress, the leaves of transgenic plants exhibited a greater decrement in ASC/DHA and GSH/GSSG ratios and ASC level and a greater increment in GSH and GSSG levels than the leaves of WT plants, but the increase in DHA level did not differ between WT and transgenic tobacco leaves. They suggested that the regeneration of GSH by GR protected plants from oxidative injury by maintaining glutathione and ascorbate redox states and the ascorbate pool. Glutathione peroxidase can use GSH to reduce H_2_O_2_ to H_2_O, with the concomitant production of GSSG [51]. Many GSTs possess GlPX activity [52]. γ-Glutamyltransferase can break down the GSH to produce glutamate and cysteinylglycine [53]. Taken together, a Mn500-induced decrement in the GSH level at P3 might be related to the decreases in the activities of APR and CS involved in GSH biosynthesis and of GR involved in GR regeneration and the increases in the activity of γGT involved in GSH degradation and in the concentration of PCs in leaves; as well as to the decreases in the activities of APR, γGCS, and CS involved in GSH biosynthesis and of GR involved in GSH regeneration and the increase in the concentration of PCs in roots (Figure 1, Figure 2, Figure 3 and Figure 4 and Table S1).

Ascorbate peroxidase uses ASC to reduce H_2_O_2_ to H_2_O with the concomitant formation of MDHA, which is reduced to ASC by MDHAR or disproportionated spontaneously to ASC and DHA. The formed DHA can be reduced to ASC by DHAR using GSH or reduced nonenzymatically to ASC by GSH itself [54,55]. Lin et al. [56] reported that *Chlamydomonas reinhardtii* Dang. cells overexpressing *CrDHAR1* displayed increased ASC recycling ability (decreased TA and DHA levels and an increased ASC/DHA ratio and ASC level) and tolerance to oxidative stress, whereas *DHAR1*-knockdown amiRNA lines exhibited decreased ASC recycling ability (unaltered TA level, decreased ASC/DHA ratio and ASC level, and increased DHA level) and tolerance to oxidative stress. They concluded that DHAR played a key role in *C. reinhardtii* cells against oxidative stress by mediating ASC regeneration. Transgenic sweet potato (*Ipomoea batatas* (L.) Lam) plants overexpressing *IbDHAR1* displayed enhanced tolerance to salt and drought stress accompanied by decreased oxidative stress and increased ASC concentration and ASC/DHA [57]. *OsDHAR1*-expressing transgenic rice (*Oryza sativa* L.) displayed less oxidative damage and increased ASC concentration and ASC/DHA under salt stress [58]. *LeMDAR* sense transgenic tomato plants displayed enhanced ASC concentration, ASC/DHA, and tolerance to oxidative stress caused by methyl viologen, and the reverse was the case for *LeMDAR* antisense transgenic tomato plants [59]. Taken together, a Mn500-triggered increase in the ASC level in leaves at P3 might be related to the reduction in the leaf DW [12] and/or the activity of APX involved in ASC utilization (Figure 3A), and Mn500-induced decrements in the ASC level in roots at P3 and in the ASC/TA in leaves and roots at P3 might be related to the decreases in the activities of GR, DHAR, and MDHAR involved in ASC regeneration and the concentration of GSH (Figure 2 and Figure 3 and Table S1).

Manganese excess can induce MG formation and accumulation [13]. In plant cells, elevated accumulation of MG can also cause oxidative injury [60]. Within cells, MG is mainly produced in the glycolytic pathway [61]. In addition to glyoxalases, GSH can also directly or indirectly participate in the degradation of MG, and the availability of GSH is associated with the removal of MG [62]. Zhang et al. [21] reported that the increase in pH reduced Cu excess-triggered increments in MG levels in ‘Xuegan’ roots and leaves by alleviating Cu excess-triggered reductions in root and leaf GSH levels, root and leaf Gly I activities, and root Gly II activity and increments in leaf Gly II activity. Yang et al. [16] observed that the increase in pH reduced Al-stimulated increases in MG concentrations in ‘Xuegan’ roots and leaves by ameliorating Al-stimulated decreases in root and leaf GSH levels and root Gly I and Gly II activities and increases in leaf Gly I and Gly II activities. The current research indicated that MG concentration in leaves and roots were significantly negatively related with GSH concentration and positively related with Mn concentration in the corresponding tissue and that MG concentrations in leaves and roots showed an increased trend with the decrease in Gly I or Gly II activity in the corresponding tissue (Figure 6 and Table S1). These results revealed that in addition to reducing Mn500-triggered MG formation possibly via glycolytic flux, the increase in pH might enhance the ability of roots and leaves to detoxify MG, thus preventing Mn500-induced accumulation of MG in these tissues. Methylglyoxal can use a GSH molecule to spontaneously convert into hemithioacetal (HTA). The formed HTA is then converted into D-lactate by Gly I and Gly II and one molecule of GSH is recycled back to the glyoxalase system, which participates in the maintenance of GSH homeostasis and subsequent scavenging of ROS [62]. Transgenic tobacco plants overexpressing *Gly I*, *Gly II*, or *Gly I*/*II* displayed higher tolerance to Zn excess. During Zn stress, the increments in the level of PCs, the sequestration of Zn in roots, and the ability to maintain glutathione homeostasis, as well as the decreases in the accumulation of MDA and MG might be responsible for Zn resilience [63]. Under salinity stress, transgenic tobacco plants overexpressing *Gly I*, *Gly II*, or *Gly I*/*II* resisted the increments in MG and MDA levels and maintained a higher GSH level and GSH/GSSG ratio [64]. For leaves, GSH concentration or GSH/TG was significantly positively related to Gly I or Gly II activity and negatively related to MG concentration except for the relationship between GSH/TG and MG concentration (*r* = −0.8345). For roots, GSH concentration or GSH/TG was significantly positively related to Gly II or Gly I activity and negatively related to MG concentration except for the relationships between GSH concentration and Gly I activity (*r* = 0.8819), between GSH/TG and Gly II (*r* = 0.9152), and between GSH/TG and MG concentration (*r* = −0.7255) (Figure 6 and Table S1). These results suggested that the increase in pH attenuated Mn500-induced increases in MG production and decreases in the activities of glyoxalases in leaves and roots, thus resisting a decrement in the GSH concentration and GSH/TG.

Reduced glutathione is involved in the MG detoxification system and ASC–glutathione cycle, serving as an interactive node between MG detoxification and the antioxidant defense system [62]. In addition to functioning in the detoxification of HMs, GSH itself and its metabolic enzymes, namely GST, GlPX, GR, γGCS, DHAR, and glyoxalases, can act additively and coordinately to prevent plants from MG- and/or ROS-induced injury [20]. Previous studies indicated that the increase in pH could decrease the accumulation of ROS, MDA, and MG and upregulate the ROS and MG detoxification systems in roots and leaves of Cu (Al)-treated ‘Xuegan’ seedlings, thereby endowing ‘Xuegan’ seedlings with Cu (Al) tolerance [16,21]. The research showed that the increase in pH reduced the impairment of Mn500 to antioxidant and glyoxalase systems and the Mn500-induced rises in MG and MDA levels in roots and leaves (Figure 7), indicating that the increase in pH enhanced the ability of ‘Sour pummelo’ roots and leaves to combat oxidative stress under Mn500. Further analysis found that the increase in pH could attenuate Cu, Mn, and Al stress-induced increases in MDA concentration and ROS (superoxide anion or H_2_O_2_) production rates in citrus roots and leaves, but the ROS and MG detoxification systems in citrus roots and leaves responded differently to Mn–pH, Al–pH, and Cu–pH treatments [16,21]. For example, the increase in pH attenuated Cu toxicity-induced increases in the concentrations of ASC, DHA, and TA in ‘Xuegan’ roots and leaves, with the exception that Cu toxicity significantly decreased root TA and ASC concentrations and did not significantly alter root DHA concentration at P3 (low pH) [16], and Al toxicity significantly increased ASC and TA concentrations in ’Xuegan’ roots and leaves, and their concentrations significantly increased with increasing pH [21]. The increment in pH lessened Cu toxicity-induced increases in the concentrations of root and leaf MTs, PCs, and TNP-SH, with the exception that Al toxicity significantly decreased the concentration of root MTs at P3 [16], and the concentrations of root and leaf PCs, MTs, and TNP-SH under Al toxicity were significantly higher at pH 3.5 and pH 4.0 than at pH 2.5 and pH 3, with the exception that the concentration of root TNP-SH under Al toxicity did not significantly differ between pH 2.5 and pH 4.0 treatments [21]. The increase in pH attenuated Al and Cu stress-induced increases in the activity of leaf SOD, with the exception that Al toxicity did not significantly alter its activity at pH 2.5, but the increase in pH prevented Al and Cu stress-induced decreases in the activities of root SOD. Also, the responses of some antioxidant enzymes and S-metabolizing enzymes in roots and leaves exhibited different responses to Mn–pH, Al–pH, and Cu–pH treatments [16,21]. The current research increased our understanding on the underlying mechanisms regarding how the increase in pH alleviates the oxidative damage caused by metal toxicity in citrus roots and leaves.

## 4. Materials and Methods

### 4.1. Plant Materials

Plant material culture and treatments were performed as described by Rao et al. [12]. Briefly, uniform 6-week-old seedlings of ‘Sour pummelo’ (*Citrus grandis* (L.) Osbeck) were transplanted to 6 L pots (two plants per pot) containing sand washed thoroughly with dilute HCl solution followed by tap water and grown in a greenhouse under natural conditions at Fujian Agriculture and Forestry University, Fuzhou (26°5′ N, 119°14′ E), with average annual sunshine hours of ∼1600 h, a relative humidity of ∼76%, and a temperature of ∼20 °C [65]. Starting seven weeks after transplanting, each pot was irrigated six times per week with freshly prepared nutrient solution containing 0.25 mM (NH_4_)H_2_PO_4_, 0.5 mM MgSO_4_, 1 mM Ca(NO_3_)_2_, 1.25 mM KNO_3_, 20 μM Fe-EDTA, 10 μM H_3_BO_3_, 2 μM ZnCl_2_, 0.5 μM CuSO_4_, and 0.065 μM (NH_4_)_6_Mo_7_O_24_ at a pH of 5 (P5) or 3 (P3) and a Mn level of 500 (Mn500) or 2 (Mn2) μM from MnCl_2_ until dripping (~500 mL per pot). The pH was adjusted by HCl. There were 16 pots (32 seedlings) per Mn–pH treatment in a completely randomized design. Twenty-five weeks after treatments, recently fully-expanded leaves and ~5 mm length root tips (one plant per pot) were harvested at noon (12:00–13:00) and immediately frozen in liquid N_2_ and then stored at -80 °C in a freezer until extraction of metabolites and enzymes. Root tips (two leaves) from a plant (one plant per plot) were pooled into a single replicate. Plants from the same plots without harvested root tips and leaves were used for the assay of SAPR. In this study, the pH levels and cultivar were chosen according to our preliminary experiment. During this preliminary experiment, we examined the effects of three pH levels (pH 3, 4, and 5) and two Mn levels (2 and 500 μM Mn) on the growth of ‘Sour pummelo’ and ‘Xuegan’ (*Citrus sinensis* (L.) Osbeck) seedlings. As the results for pH 4 and pH 5 (two cultivars) were similar, we ultimately chose one cultivar and two pH levels (pH 3 and pH5) for this study.

### 4.2. Chemicals

All chemicals used were an analytical reagent or the best commercially available grade. Disodium ethylenediamine tetraacetate (EDTA-Na_2_; analytical reagent), Triton X-100 (molecular biology grade), insoluble polyvinylpolypyrrolidone (PVPP; guaranteed reagent), ethanol (analytical reagent), HClO_4_ (guaranteed reagent), and trichloroacetic acid (TCA; analytical reagent) were purchased from Sinopharm Chemical Reagent Co., Ltd., Shanghai, China.

### 4.3. Analysis of SAPR, MG, MDA, Antioxidants, and S-Containing Compounds

Superoxide anion production rate was measured with the reduction of nitroblue tetrazolium at 25 °C [66]. Methylglyoxal concentration was determined with the N-acetyl-L-cysteine assay after extraction with 5% (*w*/*v*) ice-cold HClO_4_ on ice bath [67]. Malondialdehyde was extracted with 80% (*v*/*v*) ice-cold ethanol on ice bath and then determined by the thiobarbituric acid reactive substances assay [68].

Ascorbate and TA were extracted with 6% (*v*/*v*) ice-cold HClO_4_ on ice bath and then measured with ASC oxidase (EC 1.10.3.3, AO). Oxidized glutathione and TG were extracted with 5% (*w*/*v*) ice-cold TCA on ice bath and then determined with GR and 5,5-dithiobis-2-nitrobenzoic acid (DTNB) [66].

Total non-protein thiols and GSH concentrations were extracted and assayed according to Garg and Kaur [69]. Briefly, about 40 mg of frozen roots or leaves were ground with a precooled mortar and pestle in 1.6 mL of 5% (*w*/*v*) ice-cold TCA on ice bath. The mixture was then centrifuged at 10,000× *g* for 10 min at 4 °C. The supernatant was used for the assay of TNP-SH and GSH. For TNP-SH measurement, 0.1 mL of supernatant was mixed with 0.5 mL of reaction buffer (0.1 M phosphate buffer (pH 7.0) and 0.5 mM EDTA-Na_2_) and 0.5 mL of 1 mM DTNB. After 10 min, the absorbance was read at 412 nm. For GSH measurement, 0.5 mL of supernatant was mixed with 0.6 mL of 0.1 M phosphate buffer (pH 7.0) and 40 μL of 1 mM DTNB. After 2 min, the absorbance was read at 412 nm. The calculation formula for PCs was PCs = TNP-SH − GSH.

Metallothioneins was extracted and assayed according to Malik et al. [70]. Briefly, about 40 mg of frozen roots or leaves were ground with a precooled mortar and pestle in 1 mL ice-cold extraction solution containing 20 mM ice-cold TRIS-HCl (pH 8.6), 0.01% β-mercaptoethanol, and 0.5 M sucrose on ice bath. The extract was then centrifuged at 30,000× *g* and 4 °C for 20 min. A volume of 0.5 mL of supernatant was mixed with 0.75 mL ice-cold ethanol and 40 μL of chloroform. The mixture was then centrifuged at 6000× *g* and 4 °C for 10 min. The collected supernatant was combined with 0.5 mg RNA and 20 μL of 37% HCI and subsequently with 1.5 mL of ice-cold ethanol. The mixture was maintained at −20 °C for 1 h, then centrifuged at 6000× *g* and 4 °C for 10 min. The MT-containing pellet was washed with 87% (*v*/*v*) ethanol and was re-suspended in 75 μL of 0.25 M NaCl and 75 μL of 1 N HCI containing 4 mM EDTA. A volume of 2.1 mL NaCl (2 M) containing 0.43 mM of DTNB buffered with 0.2 M Na-phosphate (pH 8) was added to the mixture at room temperature. The sample was finally centrifuged at 3000× *g* for 5 min, and the supernatant was absorbed at 412 nm.

### 4.4. Extraction and Assay of Enzymes Associated with ROS and MG Removal

Approximately 30 mg of frozen roots or leaves were ground with a precooled mortar and pestle in 2 mL ice-cold extraction solution containing 50 mM KH_2_PO_4_-KOH (pH 7.5), 1 mM EDTA-Na_2_, 0.5% (*w*/*v*) Triton X-100, and 5% (*w*/*v*) insoluble PVPP on ice bath. The extract was then centrifuged for 10 min at 4 °C and 13,000× *g* [71]. The supernatant was used for the assay of GlPX, GST, APX, DHAR, MDHAR, CAT, GuPX, SOD, and GR activities [72]. The activities of APX, DHAR, GR, MDHAR, and CAT were determined as described by Chen and Cheng [71]. Briefly, APX activity was assayed at 290 nm in 1 mL of reaction mixture containing 50 mM HEPES-KOH (pH 7.6), 0.1 mM EDTA-Na_2_, 0.5 mM ASC, 0.2 mM H_2_O_2_, and 50 μL leaf or 100 μL root extract. The reaction was started by adding H_2_O_2_. Dehydroascorbate reductase activity was measured at 265 nm in 1 mL of reaction mixture containing 100 mM HEPES-KOH (pH 7.0), 0.1 mM EDTA-Na_2_, 0.2 mM DHA, 2.5 mM GSH, and 100 μL enzyme extract. The reaction was initiated by adding DHA. Glutathione reductase was determined at 340 nm in 1 mL of reaction mixture containing 100 mM TRIS-HCl (pH 8.0), 1 mM EDTA-Na_2_, 0.2 mM NADPH, 1 mM GSSG, and 100 μL enzyme extract. The reaction was initiated by adding NADPH. Monodehydroascorbate reductase activity was assayed at 340 nm in 1 mL of reaction mixture containing 50 mM HEPES-KOH (pH 7.6), 0.1 mM NADH, 2.5 mM ASC, 0.25 U of AO, and 100 μL leaf or 50 μL root extract. The reaction was initiated by adding AO. Catalase activity was measured at 240 nm in 1 mL of reaction mixture containing 100 mM potassium phosphate buffer (pH 6.0), 10 μL 10% (*w*/*v*) H_2_O_2_, and 10 μL leaf or 50 μL root extract. The reaction was started by adding H_2_O_2_. Glutathione S-transferase activity was measured in 340 nm in 1 mL of reaction mixture containing 100 mM TRIS-HCl (pH6.5), 1 mM 1-chloro-2,4-dinitrobenzene (CDNB), 1.5 mM GSH, and 100 μL enzyme extract. The reaction was initiated by the addition of CDNB [73]. Superoxide dismutase activity was measured at 560 nm using a photochemical assay system consisting of riboflavin, methionine, nitroblue tetrazolium, and enzyme extract. One U of SOD activity is defined as the amount required to produce a 50% inhibition of nitroblue tetrazolium photoreduction [74]. Guaiacol peroxidase activity was determined at 470 nm in 1 mL of reaction mixture containing 100 mM potassium phosphate buffer (pH 6.0), 16 mM guaiacol, 5 μL 10% (*w*/*v*) H_2_O_2_, and 20 μL leaf or 10 μL root extract. The reaction was started by adding enzyme extract [75]. Glutathione peroxidase activity was assayed at 340 nm in 1 mL of reaction mixture containing 100 mM potassium phosphate buffer (pH 7.0), 1 mM NaN_3_, 1 mM EDTA-Na_2_, 2 mM GSH, 0.12 mM NADPH, 1 unit GR, 0.6 mM H_2_O_2_, and 20 μL leaf or 50 μL root extract. The reaction was started by the addition of H_2_O_2_ [19].

About 30 mg of frozen samples were extracted with 1 mL of ice-cold 100 mM TRIS-HCl (pH 8.0) containing 10 mM EDTA-Na_2_, 2 mM dithiothreitol (DTT), and 4% (*w*/*v*) insoluble PVPP on ice bath. The extracts were then extracted at 4 °C and 13,000× *g* for 10 min. The resultant supernatants were used immediately for the measurements of CS, SiR, APR, γGT, γGCS, ATPS, Gly I, and Gly II [75]. Cysteine synthase was measured as described by Warrilow and Hawkesford [76]. Enzyme extract (20 μL) was incubated at 25 °C for 10 min with 0.2 M TRIS-HCl (pH7.5), 10 mM DTT, 3 mM Na_2_S, and 5 mM O-acetyl-L-serine (OAS) in a total volume of 0.8 mL. This reaction was initiated and terminated by the addition of OAS and 0.2 mL of 1.5 M TCA, respectively. The resultant cysteine was assayed by the ninhydrin method. Sulfite reductase activity was assayed at 340 nM in a mixture (1 mL) of 10 mM TRIS-HCl (pH 7.5), 0.1 mM EDTA, 0.2 mM NADPH, 0.5 mM Na_2_SO_3_, and 100 μL enzyme extract [75]. Adenosine 5ʹ-phosphosulphate reductase activity was determined in a mixture (1 mL) of 50 mM TRIS-HCl (pH 8.0), 4 mM Na_2_SO_3_, 0.5 mM K_3_Fe(CN)_6_, 8 mM EDTA-Na_2_, 0.4 mM AMP, and 100 μL enzyme extract. The reaction was started by the addition of enzyme extract. The decline in absorbance was recorded at 420 nm against a blank containing the mixture without enzyme extract [75]. γ-Glutamyltransferase activity was measured according to Chen et al. [75]. Enzyme extract (100 μL) was incubated at 30 °C for 30 min with 100 mM TRIS-HCl (pH 8.0), 20 mM glycylglycine (Gly-Gly), and 2.5 mM L-γ-glutamyl-p-nitroanilide in a total volume of 1 mL, then the reaction was stopped by adding 1 mL of 25% (*w*/*v*) TCA. The resultant p-nitroaniline (ε = 1.74 mM^−1^ cm^−1^) was measured at 405 nm. γ-Glutamylcysteine synthetase activity was assayed in a mixture (1 mL) of 100 mM TRIS-HCl (pH 8.0), 150 mM KCl, 20 mM MgCl_2_, 2 mM EDTA-Na_2_, 0.2 mM NADH, 10 mM α-aminobutyrate, 10 mM glutamate, 5 mM ATP, 2 mM phosphoenolpyruvate (PEP), 10 U of lactate dehydrogenase (EC 1.1.1.27, LDH), 7 U of pyruvate kinase (EC 2.7.1.40, PK), and 100 μL enzyme extract [75]. ATP sulfurylase activity was measured according to Chen et al. [75]. Enzyme extract (100 μL) was incubated for 15 min at 37 °C with 80 mM TRIS-HCl (pH 8.0), 2 mM Na_2_ATP, 5 mM Na_2_MoO_4_, 7 mM MgCl_2_, and 0.032 U mL^−1^ of sulfate-free inorganic pyrophosphatase in a total volume of 0.6 mL. The reaction was initiated and stopped by the addition of enzyme extract and 2 mL of 20% (*w*/*v*) TCA, respectively. The resultant phosphate was determined as given by Ames [77]. The blank contained the same mixture and enzyme extract without Na_2_MoO_4_. Glyoxalase I activity was determined in a reaction mixture (1 mL) of 100 mM potassium phosphate buffer (pH 7.0), 15 mM MgSO_4_, 1.7 mM GSH, 3.5 mM MG, and 100 μL enzyme extract. The reaction was started by the addition of MG. The increase in absorbance was recorded at 240 nm (extinction coefficient of 3.37 mM^−1^ cm^−1^) against a blank containing the reaction mixture without enzyme extract [75]. Glyoxalase II activity was measured by monitoring the production of GSH at 412 nm (extinction coefficient of 13.6 mM^−1^ cm^−1^) and 25 °C in a reaction mixture (1 mL) of 100 mM TRIS-HCl (pH 7.2), 1 mM S-D-lactoylglutathione (SLG), 0.2 mM DTNB, and 100 μL enzyme extract [75].

All ROS and MG scavenging enzyme activities were measured at 25 °C, except for the specified temperature.

### 4.5. Statistical Analysis

Principal coordinate analysis was performed with ChiPlot (https://www.chiplot.online/ (assessed on 7 June 2025)). Pearson’s correlation coefficients for mean valves (*n* = 4) were calculated with the SPSS statistical software (version 17.0, IBM, Armonk, NY, USA). A Bonferroni-corrected paired *t*-test was performed for pairwise comparisons. Means were tested by two-way ANOVA followed by the LSD at *p* ≤ 0.05 using DPS 7.05 (Hangzhou Ruifeng Information Technology Co., Ltd., Hangzhou, China).

## 5. Conclusions

The current research indicated that the increase in pH attenuated Mn500-induced increases in SAPR and MG and MDA concentrations and alterations of ROS and MG detoxification systems in ‘Sour pummelo’ leaves and roots, suggesting that the increase in pH enhanced the capability of these tissues to detoxify ROS and MG under Mn excess, thereby contributing to less oxidative damage. Therefore, the current findings provided new information on the roles of both ROS and MG removal systems in the augmented pH-mediated amelioration of oxidative injury in roots and leaves caused by Mn excess. Notably, this experiment was conducted using sand-cultivated seedlings, not adult trees grown in field soils. Soils contain humus and a large number of microorganisms. Humus can reduce HM phytotoxicity via chelating HMs [78]. Soil microorganisms can produce and release organic acids, amino acids, and glomalin and lower bioavailable HMs in soils [79,80]. Sand experiments may overestimate the phytotoxicity of Mn in field soils due to the influences of soil microorganisms and humus. Additionally, in natural soils, pH can affect not only Mn availability but also root microbial interactions, organic acid production, and cation exchange dynamics. In order to develop soil amendments using lime and other alkaline substances to protect citrus trees from oxidative damage caused by excessive Mn, further experiments need to be conducted on adult citrus trees grown in field soils.

## Data Availability

Data are archived in L.-S. Chen’s lab and available upon request.

## Supplementary Materials

The following supporting information can be downloaded at https://www.mdpi.com/article/10.3390/plants15010172/s1, Table S1: Pearson’s correlation coefficient matrix for the mean values of 64 physiological parameters in four Mn–pH treatments.
